# “What? That’s for Old People, that.” Home Adaptations, Ageing and Stigmatisation: A Qualitative Inquiry

**DOI:** 10.3390/ijerph16244989

**Published:** 2019-12-08

**Authors:** Cathy Bailey, Dominic Aitken, Gemma Wilson, Philip Hodgson, Barbara Douglas, Rachael Docking

**Affiliations:** 1 Nursing, Midwifery and Health, Coach Lane Campus, Northumbria University, Newcastle upon Tyne NE7 7XA, UK; gemma.wilson@northumbria.ac.uk (G.W.); philip2.hodgson@northumbria.ac.uk (P.H.); 2 School of Architecture Planning and Landscape, Newcastle University, Newcastle upon Tyne NE1 7RU, UK; dominic.aitken@newcastle.ac.uk; 3 Elders Council of Newcastle, Newcastle upon Tyne NE4 5PL, UK; barbara.douglas@elderscouncil.org.uk; 4 Centre for Ageing Better, National Charity, London EC1V 4AD, UK; rachael.docking@ageing-better.org.uk

**Keywords:** home adaptations, housing, older people, ageism, healthy ageing, independent living

## Abstract

Older people, even those living with long-term conditions or poor mobility, can be supported to live well at home, through adapting their home to meet changing need. Installing home adaptations, from grab rails to walk in shower rooms, is cost effective, may prevent falls, reduce social isolation and improve self confidence. Despite austerity cuts to public spending, the UK government increased home adaptations’ funding. However, not much is known about older people’s experiences and understanding of acquiring and living with home adaptations and uptake of home adaptations could be improved. Using wearable camera and face to face interview data, this qualitative study explored a diverse group of older people’s retrospective experiences (*n* = 30). Focus group discussions were also carried out with a wide range of professionals involved in the provision of home adaptations (*n* = 39). Findings suggest people may delay having adaptations, because of perceived stigmatising associations with decline and vulnerability. As delaying the installation of home adaptations until crisis point is known to reduce their effectiveness, such associations need to be challenged.

## 1. Introduction

Over 90% of those aged 65 and over and living in the UK, live at home [1]. Many such older people, enjoy independent and fulfilled lives. However, the home environment may not be conducive to changing need. The 2016 Health Survey for England (HSE) found that, for those aged 65 and over, 31% of women and 24% of men required help with at least one activity of daily living, such as eating, washing and dressing [2]. Some 2.4% of UK citizens are aged over 85 [3] and whilst many live at home, 29 per cent of people aged 85 and older live in substandard housing [4]. In 2014, a household survey highlighted how 59% of disabled people who are 65 and over and living in the UK report that, within five years, they will need accessible housing features [5]. 

The UK housing stock is one of the oldest in Europe and a lot of older homes were not constructed to modern accessibility standards. The Building Research Establishment has calculated that poor housing costs the English National Health Service £1.4bn annually [6]. There also appears to be little affordable housing being built in locations with reliable transport links and accessible local facilities. People do not necessarily have the choice to move to more accessible homes [7]. Despite high levels of home ownership, it has also been estimated that 67 per cent (1.1 m people) of older people living in poverty are owner occupiers [8]. This may be partly due to the 1980s UK Prime Minister, Margaret Thatcher Government’s affordable “right to buy” council home schemes which enabled tenants to purchase their homes at a discount. In more deprived areas and with little or no available equity such homeowners are unable to exercise housing choice or put in place, repairs and adaptations [5]. 

Accessible and affordable housing is critical to ageing well in a place of one’s choosing, yet the UK’s lack of such housing is echoed in other countries with ageing populations. In the United States, for example, Stone [9] asserts an urgent need for a comprehensive national senior housing policy agenda and implementation strategy to address an historical lack of government investment in and thus shortage of, affordable and accessible housing for low-income older adults and individuals living with disabilities. In 2016, the European Anti-Poverty Network [10]( p.41) highlighted lack of accessible and affordable housing for “vulnerable groups”, including older people, as a priority challenge to tackling health and social inequalities and from with a number of European countries, highlighted how “living alone in houses with poor accessibility contributes [to older people’s] isolation and exclusion”.

However, older people, even those living with long-term conditions or poor mobility, can be supported to live well at home and to maintain social connections, through adapting their home to meet changing need. A review of the home adaptations international literature [11] and a later review [12] find compelling evidence that minor home adaptations, for example adjusting lighting, or installing hand rails in bathrooms, are a good intervention for preventing falls and injuries and also cost-effective. These reviews also point to minor adaptations working best when in conjunction with necessary home improvements and repairs, such as removing falls and trip hazards. Care & Repair England [13], a national charity that seeks to improve the homes and living conditions of older people, estimated that 300 older people can be helped by a handyperson service, thus dealing with minor repairs such as securing loose carpeting, for the same cost of one place in a care home for a year (£30,000 vs. £100). There is some evidence that, in some circumstances, major adaptations, such as a stair lift or walk in shower, may support people to remain at home. However, the overall evidence on the effectiveness of major adaptations is less robust than that for minor adaptations [12]. 

Despite austerity driven central government budget cuts to local government, the UK Government continues to increase its home adaptations funding allocated to local authorities [14]. This has increased from £220 million in 2015/2016 to £468 million in 2018/2019 and expected to rise in 2019/2020 [4]. A Care & Repair England report identified some high-quality and innovative practice in the provision of home adaptations for older people [15]. The authors also note however that quality of provision is “highly variable” across the UK and only 7% of UK homes meet basic national accessibility requirements. Overall provision still appears to be patchy across local government and there is lack of a unified approach to monitoring outcomes. 

Within the last ten to fifteen years, there has been some rich, small scale, qualitative research that explores older people’s perspective and meaning of home adaptations in relation to supporting independent living at home [16,17]. For example, a 2008 Australian, qualitative study explored home modifications in relation to older people’s understanding of home as a dynamic, physical, social and personal space suffused with meaning and how having home modifications may impact on such understanding [16]. Aplin, de Jonge and Gustafsson [17] also focused on meaning making to qualitatively explore older people’s home modifications’ decision making, to better inform occupational therapists. 

This paper adds to this qualitative focus by reporting on a small part of a qualitative study that focused on both the self-reported experiences of 30 older people acquiring and living with home adaptations and on the findings from four focus group discussions (*n* = 8–13), with 39 professionals involved in the provision of home adaptations. The overall findings from the study are reported upon elsewhere [18,19,20]. Here, we report in more detail on an overarching finding raised by older adults and practitioners, that delaying having home adaptations until a person was “struggling”, was partly to do with perceived negative associations of ageing, with loss of independence and vulnerability and also because of their medicalised appearance. 

## 2. Methodology

### 2.1. Research Design

Qualitative research was commissioned by Centre for Ageing Better, a national and independent charitable foundation to explore motivations for and barriers to acquiring home adaptations and their impact on quality of later life. Subsequently, led by a team from Northumbria University, in the northeast of England, in partnership with two contiguous local authorities, Newcastle University and an older persons’ forum, a qualitative study was designed and carried out to explore a diverse group of older people’s retrospective experience of living with home adaptations (*n* = 30). This formed Strand 1 of the study and included six of the thirty participating older people, using a wearable camera (Phase 1 of Stand 1) and all thirty undertaking a semi-structured, home based interview (Phase 2 of Strand 1). Strand 2 of the study involved focus group discussions with a wide range of professionals with a role in the provision of home adaptations (*n* = 39) and seeking best outcomes for home adaptations’ users. Ethical approvals were sought and granted from the Health and Life Sciences Ethics Committee, Nursing Midwifery and Health Department (Reference: 4217) Northumbria University UK. 

With a focus on understanding and describing lived experiences [21], this study adopted a phenomenological approach to methodology. Similar to that of Husserl, van Manen’s hermeneutic phenomenology, seeks to understand human experience as it is lived. Whilst Husserl spoke of a “life world” to stress the immersiveness of everyday lived reality, van Manen also refers to Heidegger’s, “Daisein” or “Being-there” to capture how human beings are involved with, act and exist in the world [22].

### 2.2. Sampling and Recruitment 

For Strand 1 and to capture diversity, a purposive sampling profile was designed and included: minor or major home adaptation and its funding source; age range (65–74, 75–84, and 85+), ethnicity, gender, household composition and house type and tenure. Using this sampling profile, each of the participating local authorities led the recruitment within their location (*n* = 15 from each site) focusing on people aged 65 and over, who had accessed the local authority’s home adaptations services within the last two years. The voluntary older persons’ forum, works with agencies, charities, organisations and individuals, to ensure the views of the over-50s are taken into account by decision-makers in the region, supported recruitment across the two sites, using their considerable knowledge of where people go for information, from local support groups, to national and local charities, to word of mouth. This supported capturing a range of the sampling profile as described above and Table 1 illustrates this breadth for the thirty participants who took part in the study.

Strand 2 of the study captured practitioner and professional experiences of providing home adaptation related support and services, through four focus groups (two at each of the local authority study sites (*n* = 8–13)) carried out with 39 practitioners. These included personnel and volunteers from charities, support and signposting agencies, as well as occupational therapists, community nurses, home adaptations local authority service providers, contractors, builders and suppliers and a home adaptation company. Sampling focused on encompassing the breadth of practitioners involved in the provision of home modifications and also ensuring such breadth across the two participating local authority study sites. Recruitment drew on the study’s partnership contacts, considered and approved by the study steering group.

### 2.3. Data Collection

Over six months, participants’ lived experiences were captured within this two-strand approach. Strand 1 involved semi-structured interviews carried out with older adults in their own homes (15 participants from each local authority location). The semi-structured interviews were informed by initial work carried out using a wearable camera [18]. From the cohort of 30, six (three from each local authority location) chose to use a wearable camera for a day in their own home, as well as participate in a semi-structured interview. The wearable camera captured every day, taken for granted, interaction with and use of, home adaptations. Such interaction may not be recalled within an interview but captured images may then act as prompts, triggering the participant to recall habitual behaviour. We discuss this in detail elsewhere [18]. During a semi-structured interview carried out in these participants’ homes, the images were reviewed and confidentially discussed with each participant. The findings from the camera footage informed the subsequent interviews. Interview questions explored participant reasons and triggers for pursuing home adaptations and sought to understand their prior every day routines and activities, experiences of and routes to, accessing home adaptations, being assessed for eligibility (financially in relation to available grants and health status), the installation period and immediate and ongoing impacts of the adaptations. 

Strand 2, practitioner focus group discussions with a total of 39 practitioners, involved two focus group discussions at each of the two participating local authority sites (*n* = 4) with 8–13 participants attending each focus group. These lasted approximately two hours with refreshments. Discussion questions mirrored those used with the older people’s interview schedule (triggers for pursuing home adaptations, referral routes, assessment processes (financially in relation to available grants and health status), installation practices, procedures, evaluation and known longer term outcomes). Permissions were sought and granted to audio record the discussions. 

### 2.4. Data Analysis

Face-to-face interviews and focus group discussions were transcribed verbatim and entered into NVivo qualitative analysis software. Data were analysed by the research team using open and axial coding. Codes were drawn together to identify overarching themes. Older persons’ forum members supported analysis to ensure accessible language and real-world relevance. Following analysis, to test and refine the emerging themes and recommendations and add additional rigour, a range of stakeholders, including those who attended the focus group discussions were invited to an interim findings and evaluation event hosted by Housing Learning and Improvement Network (Housing LIN). Housing LIN includes housing, health and social care professionals in England, Wales, and Scotland, working together to promote and realise innovative housing solutions for older people.

## 3. Findings—Ageism and Home Adaptations

Practitioners spoke of triggers signalling disability and clinical aesthetics (appearance and utility). Older adults presented diverse personal contexts and understanding of ageing, appearance and perceptions of disability and adapting to and with the adaptation. 

### 3.1. Triggers Signalling Disability 

As these extracts from one of the focus group discussions illustrate, practitioners identified common “triggers” for installing home adaptations:

*A lot of the referrals come through from the care agencies. That are going in to see that person to provide care and the older person themselves has not been aware that there’s been something wrong or… They need…*

(National Health Service {NHS} Nurse, Focus Group {FG} 3)

Charities supporting older adults with specific conditions, such as a visual impairment, may also raise the need for home adaptations:

*Sometimes our service users have other disabilities, besides their visual impairment. And, also, another one that seems to come up fairly often is, you know, can we have things marked up differently. Such as steps, hand rails–painted in bright colours, so that people can see them and know that they’re there. That gives them confidence to move around more freely.*

(Vision Charity worker, FG 3)

Word of mouth may also raise awareness of adaptations:

*And often older people might have experience of a friend or a neighbour who has received a piece of equipment. They might have been struggling, but might not have realised that there might be a solution to that. And then they sort of realise there might be something out there that can help me as well, so…*

(Technical Assistant, Adaptations, FG 3)

Whilst some people self referred, there was also agreement across the four focus groups that there is a general lack of awareness of how to access home adaptations:

*People actually don’t know that these services are out there. And also how to access them. You don’t get taught, at any point in your life, how to become an older person. It just sort of happens, [. . .] You know, if you have a child,... you’ve got your health visitor and they explain what you’re supposed to do. You become old and no-one is there telling you.*

(Age related Charity, FG 1)

Occupational therapists cited common reasons for choosing to seek home adaptations:

*I suppose it’s anything that stops somebody doing the things that they want to do within their own homes. So, that’s not always an adaptation as we know. And we do have a process that we go through, that if equipment won’t meet those needs, then we might proceed to an adaptation. There are all sorts of, sort of, avenues that we need to go through first. Before we reach the adaptation stage.*

(Occupational Therapist {OT}, FG 1)

*Typically asking for a grab rail. [. . .] Or they might have been in hospital, and they’re wondering how they’re going to cope when they get back out of hospital.*

(OT, FG1)

There was also recognition that there may be reluctance to accept the adaptation:

*You also get a lot of people who don’t want adaptations, and they will struggle on and...[. . .] It’s change. [. ..] [T]he change in the house and leaving the house to... To family. .*

(Home Adaptations Grants Officer, FG 4)

Practitioners questioned whether some of the reluctance may be to do with wider cultural associations of adaptations with negative stereotypes of ageing and vulnerability:

*You walk down the street, and the street says, “Vulnerable older person. Vulnerable older person.” A key safe or grab rails or the ramps or whatever they happen to be.*

(Age related forum, FG 3)

There was discussion about how such negative associations between adaptations and vulnerability, might lead older people to delay acquiring adaptations until a point of crisis:

*They often say that they’re giving in. And that’s the way they look at it. Is that they are giving in to whatever is wrong is wrong with them. Whereas we try... Like [Name] [00:11:49] said, we try to say that it’s not. We’re just encouraging your independence, and take that tack with them.*

(Community Nurse, FG 1)

*I think they see it as almost a reduction in their independence if equipment is being suggested to go in to help with this and this. And I think they find that very difficult to say, well, actually, what we are trying to do is promote your independence and try and keep you safe with this equipment. [. . .] They view it in a different way to that. [. . .] it’s that withdrawal of their independence, rather than trying to promote their independence.*

(OT, FG 1)

### 3.2. Clinical Aesthetics

Across the focus groups, there was agreement that the design of equipment offered can be quite utilitarian, generally white rails, tiles and fittings:

*And I think often, in many situations, they don’t want to do any major changes to their home. It might influence how it will look, how they maybe are passing it on to their family as an inheritance. And, sometimes, you know, as we all are, you don’t want to make your home look too clinical. And I think that’s the challenge for us.*

(OT, FG 4)

Practitioners shared experiences of older people disliking the clinical appearance of grab rails and aids in the bathroom such as a fixed seat in the shower, as they remind them of a hospital environment or do not fit the existing home décor:

*With the best will in the world, adaptations can, you know, provide a very clinical... a more clinical environment, as assessed by need. And it’s trying to have that.... That, sort of, conversation with them about what is in their best interest, really. To keep them in the home, safe. And I think you have to be very sensitive to that.*

(OT, FG 4)

*An adaptations manufacturer suggested that there are more aesthetically pleasing products but that “there’s a cost implication”.*

(Adaptations Manufacturer, FG 3)

### 3.3. Diverse Personal Contexts and understanding of Ageism

Each participant perceived and prepared for ageing in different ways. Whilst some talked about the need for forward planning, others appeared to accept dealing with change as “it happened”, as one got older. Others expressed not wishing to ask for help for fear of signalling being old:

*“I wouldn’t have dreamed of having anything like that [home adaptation], you know. What? That’s for old people.”*

(Participant 04)

Other participants were more sanguine:

*I fell, and I was lying there and I pressed my button [pendant alarm]. They came, let themselves in. Two minutes later, I was up. [. . .] So, you know... Life is what it is. There’s nothing you can do about it.*

(Participant 24)

For another participant, giving up bathing was compensated by a replacement shower affording more independence:

*I mean, when I was working I... I mean, I used to enjoy the bath. But I enjoy the shower better now. And I think it’s knowing that I’m going in there and I’ve got my own independence. That’s... That’s the big thing. When you... Like, you know, I push myself to do what I can. [. . .] I want to do what I can do, you know.*

(Participant 32)

A fear of hip replacement surgery left one participant dealing with a lot of pain, having to travel to a family member home to use a shower and enormous difficulty with stairs:

*Like, I had to go up sideways. And when I got, like, halfway up, I had to turn around and go up backwards. And it was very dangerous, the way I was doing it*

(Participant 09)

*Eventual hip surgery, a shower and a stair lift has “…. It’s just improved my life a hell of a lot more”*

(Participant 09)

Alternative living arrangement such as a residential home or supported living, were seen to signal becoming “dependent” and losing quality of life:

*“When you see these old people’s homes, and their whole... Chairs around a television set. Oh, God spare me that. Really, I think that’s... That must be the end, when you’re reduced to that kind of thing.”*

(Participant 23)

One participant, who recently moved into one of a group of local authority bungalows, these supported by an on site warden who regularly visits, commented on leaving a three bedroom family home with their spouse and moving to a one bedroom bungalow, *“[w]hen you’re downsizing you’ve got to get rid of... We got rid of so much stuff, it was unbelievable, wasn’t it? [. . .]* (Participant 17)

### 3.4. Appearance and perceptions of age and disability

Home adaptations were considered to alter the appearance of home, an aesthetic that was not pleasing. External to the house handrails to aid entry and exit were particularly disliked:

*“I really would have struggled to get in. Because there wasn’t a handle. And I don’t want a handle at the front door. Because I don’t like the look of it [a grab rail]. It’s like a pipe... A bit like a sewage pipe, you know what I mean?”*

(Participant 30)

Others however acknowledged that that they had been offered some choice in the design of an installed shower unit, particularly some colour choice for tiles and flooring: “*It looks really nice. Everybody remarks on it when they come in. They’ll say, Oh, it’s really nice. Uh-huh. The blue floor - it’s nice.”* (Participant 17). 

Other participants, whilst voicing opinions on not liking the very functional look of home adaptations, commonly grab rails, but also larger adaptations such as stair lifts, also acknowledged their benefits and that with time, even a functional looking home adaptation can become an accepted part of their home:

*“That was one of the reasons that I didn’t want it. Because... Because of the look of it. But then you’ve got to weigh up the benefits, and the benefits outweighed the... You know, you’ve got to forget about sort of the look of things and think what benefits it’s given you, you know. And now, I never notice... You never notice it.”*

(Participant 16)

### 3.5. Adapting with and to the Adaptation

Some participants accepted adaptations as simply a reality of being an older person. One participant stressed that, to remain in one’s own home, older people had to accept and adapt to home adaptations, *“[w]hether you want to or not”* (Participant 04).

Another participant had suffered a stroke and recounted deteriorating mobility particularly at exit and entry points to their home, “*Well, I would say I was going outside less, because I was frightened for the steps and that. Going down them. I was a bit unsteady on my feet.”* (Participant 51). Subsequently, having hand rails installed at these strategic points had increased their confidence:

*Well, I feel when I go out in the back [garden/yard], I’m more confident. I’ve got something to hold on to. It’s the same at the front. You know, going down the step, I’ve got something I can hold on. And if I come in and I’ve got heavy shopping and that, it helps me.*

(Participant 51)

Although the same participant also conceded that some adaptations had their limitations. Whilst installing a second bannister on the stairs improved getting up the stairs, as previously, *“Well, I had been up on my hands and knees. On the stairs”,* having two bannisters also meant: “*[n]ot very good. I mean, I still can’t carry stuff up and down the stairs. Because I’ve got to hold onto them*.*”* However, when considering the next stage of adaptation such as installing a star-lift, *“[a] the moment, I’m alright with my two… As long as I have my two bannisters, I’m alright. I can manage up and down”* (Participant 51). 

Another participant was unequivocal in their support of installing home adaptations that met changing mobility or health needs and promoted continued independence:

*100% get it done. It does change your life. Which, I’ll say, I’m a lot cleaner now to what I was. I’m a lot more independent in the house and everything else, so... Really, yes, 100%, get it done.*

(Participant 09)

For participants and following installation of an adaptation, overall use varied. For some and within a year of having an adaptation installed, it was taken for granted. For example, a stair lift “*... I’ve got so used to it”* (Participant 30) or a walk in shower, was *“just ideal to be able to have a shower every day” which also having good instant heat, “relieves the pain” of “aching joints”* (Participant 22). Others used their adaptation less, depending on how they were feeling and the extent to which they required support:

*“I never know from one [day] to another what I’m going to be like. So I keep saying to [my husband] I’m going to walk up the stairs to try and keep my fitness...Because I sit around far too much and I try...to do as much as I can.”*

(Participant 03)

One participant reflected on how adaptations and equipment are unlikely to provide solutions to the root cause of needing such adaptations, such as restricted mobility or poor balance:

*“I’ve got a trolley [walking frame with wheels] to get around, but in fact my mobility is restricted. And…you want to get something…[but] you don’t want to have to reach over and get the trolley, you really want to…reach over and get something straightaway…So you have to live within your limitations. They...modify the limitations, but they don’t remove them.”*

(Participant 07)

## 4. Discussion

Given the encouraging evidence base and in the UK, as in other countries with ageing populations, the shortage of adaptable and accessible homes, then home adaptations should be part of a success story of ageing well in homes and communities. However, our “lived experiences” study findings suggest that, alongside the need to address the “highly variable” home adaptation provision in the UK (albeit whilst acknowledging some high-quality and innovative practice) [15], there is a need to tackle “ageism”. Robert Butler, a psychiatrist, coined the term ageism to denote prejudicial and discriminatory behaviour towards older people, perpetuated through stereotyping [23]. 

Both practitioner and older adults’ findings explicitly and implicitly illustrate nuanced, age related, stigmatising and stereotyping barriers to the installation and use of home adaptations. Responses were varied. A few participants expressed initial reluctance at having home adaptations installed because of “the look of it” and then decided that benefits to quality of life outweighed such concerns. Others seemed fatalistic, accepting the need for change as “it happened”, with ill health and limited mobility, an inevitable part of ageing. A few wished to forward plan. Others were afraid to “appear old” or signal frailty and decline, by asking for help. Whilst illustrating a diversity of individual experience of and response to, ageing, all practitioners and participants seemed to share a common understanding of “ageist attitudes”. 

It has also been recognised that ageism may be internalised by older people. Older adults may perceive old age negatively and hold negative views about those older or more disabled than themselves [24]. However, within our study, older people did not appear to internalise ageism. Rather they expressed awareness of “ageist” connotations associated with needing home adaptations, as with the participant who said, “You sort of think of two stair rails as a sign of old age. A sign of incapacity” [Participant 16]. Such ageist understanding of the installation of a neutral object, such as a second stair rail, into an older person’s home, may in itself perpetuate negative attitudes to the ageing process [25]. 

In 2009, the Centre for Policy on Ageing (CPA) was commissioned by the Department of Health (DH), to conduct a review of ageism and age discrimination in social care in the UK. This identified a “legacy of historical ageism”, defined by age differentiated services. There was also a “low level of expectation by older people of what they will receive from services and providers of services limited views of what is acceptable to older people in terms of quality and choice compared to the population as a whole” [26] (p. 45). From within this study and ten years on, from this review, that a participant should remark: “*You know, you’ve got to forget about sort of the look of things and think what benefits it’s given you, you know”* (Participant 16) is disappointing. Rather we would suggest, given the evidenced, positive impacts that home adaptations may play in supporting independent living, they should be both beneficial and “have a look”, that is neither better placed within a clinical environment nor is perceived in a negative way. Rather, it simply “is” part of the success story of ageing.

There are also ongoing calls to address ageism. Recently, Walker, an eminent social gerontologist, called for a “radical new strategy on ageing”, with social policy endorsing and promoting “active ageing” across the life course [27]. This is to acknowledge how life experiences may be socially as well as biologically constructed. As Walker [27] (p. 1) says, “while ageing is inevitable, it is also plastic”. He points to a substantial body of research, including the UK 10-year “New Dynamics of Ageing“ multi-disciplinary collaborations programme (2005–2015) and prior and successive UK and international research that supports: “a dynamic process of more or less continuous change in both ageing and the meaning of later life” [27] (p. 5).

Mitigating risk factors across the life course may modify the ill effects of chronic conditions, such as diabetes and limited mobility that can negatively impact the quality of later life. Such changes do not need to be an “inevitable part of ageing”. This is also supported by recent research by Gore et al. [28] (p. 764) suggesting that we have been relying on aggregate scores of activities of daily living, to measure functional decline as we age. Rather the authors propose a framework (Compression of Functional Decline) that enables us to work with the malleability of a “realistic view of age-related functional decline in the context of modifiable behaviour to counter widespread public misconceptions about ageing and inform improvements”. 

Working from within the context of modifiable behaviour [28] and with malleable ageing [27] may go some way to decouple the unhelpful association of home adaptations with negative and stigmatising portrayal of ageing. Rather than home adaptations being part of a story of decline and increased dependency, accessible, attractive and affordable adaptations should be available in response to a positive life choice about when “a little bit of help” is likely to promote optimum quality of life. Indeed, Gore et al.’s [28] research and based on a rich dataset of real time assessments, ADL LifeCurve™, has produced an online tool developed by ADL Research and Newcastle University’s Institute for Ageing [29] that can map difficulties with daily tasks (functional decline), such as cutting toe-nails or getting up from the floor, to plan and deliver the best of timely services and supports. These in turn may prevent further deterioration, improve current capability and potentially, overall quality of life. This “optimising” current capability was echoed by some of the study participants, who welcomed an adaptation but wished to remain fit. A participant with a stair lift, who had variable days but “better” days meant maintaining fitness by walking up the stairs (Participant 03). 

Home adaptations should be a positive choice for supporting independent living at home but we also acknowledge there are deeper challenges. Walker [27] asserts that, whilst evidence suggests that, in the 2000s, healthy life expectancy (HLE) increased more than life expectancy (LE), social determinants of health may impact on who gains the most. Evidence demonstrates how the social class gradients for HLE and LE rates are overall higher within the managerial and professional classes [30,31]. Walker also notes research by Jagger [32] that points to lower HLE being linked to area deprivation, black and minority ethnic status and low incomes and a European study that associates lower HLE for men and women with material deprivation [33]. 

Product designers, service providers, users and others need to consider lifetime ageing, “a dynamic process of more or less continuous change in both ageing and the meaning of later life” [27] (p. 5). This is so that adaptations, equipment, services and supports, designed to optimise good living in places of our choosing, are not just for “old people” but rather, can adapt to changing need. They should also be neutral, simply an aid to optimum independent living, at all ages. Relatedly, we also tentatively suggest that our coupling of our findings that explicitly and implicitly illustrate nuanced, age related, stigmatising and stereotyping barriers to the installation and use of home adaptations, with “ageism”, is relatively novel. Whilst we are aware of some early discussion on ageism and its potential negative impact on older people’s access to environmental interventions, such as home adaptations (see, for example, [34]), there seems to be a need for more overt challenging of ageism and its potential to negatively impact on promoting home adaptations as neutral and effective support for independent living. 

## 5. Limitations

The study has a small sample; one English, geographical location; retrospective data collection, mostly a year, for a few, almost two years, after installing home adaptations; and privileges a home adaptations journey, as recalled by these participants. Moreover, the older people who participated had chosen to install home adaptations. More needs to be known about those who do not consider home adaptations. Some of these limitations are offset by the study’s strengths: our use of participant wearable cameras to elicit not just what was told but also what was experienced and inclusion of professional “lived experiences” of providing home adaptation services and supports. 

## 6. Conclusions

Our qualitative findings both confirmed and in some instances refuted, underlying ageist attitudes, to be found from within participants’ recounted, lived experiences of having home adaptations. Both provider and user of such services shared common understanding of how the need for such adaptations, may be negatively perceived by others and older people themselves. Whilst the evidence is clear on the potential benefits and positive impacts that home adaptations can have, our findings suggest that people may not be making a choice to adapt their home early enough, because of the negative associations between ageing signifying decline and adaptations. The lack of attractive and inclusive products seems to entrench this.

## Figures and Tables

**Table 1:** Profile of Participants.

Variable		Site 1	Site 2
Age	65–74	17% (*n* = 5)	7% (*n* = 2)
	75–84	23% (*n* = 7)	27% (*n* = 8)
	85+	10% (*n* = 3)	17% (*n* = 5)
Gender	Male	27% (*n* = 8)	17% (*n* = 5)
	Female	23% (*n* = 7)	33% (*n* = 10)
Ethnicity	White British	50% (*n* = 15)	43% (*n* = 13)
	Other	0% (*n* = 0)	7% (*n* = 2)
House Type	Bungalow	17% (*n* = 5)	0% (*n* = 0)
	Semi-detached	20% (*n* = 6)	23% (*n* = 7)
	Terrace *	7% (*n* = 2)	20% (*n* = 6)
	Flat/Apartment	7% (*n* = 2)	7% (*n* = 2)
Tenure	Social rent (local authority)	17% (*n* = 5)	7% (*n* = 2)
	Social rent (housing association)	3% (*n* = 1)	3% (*n* = 1)
	Owner occupier	23% (*n* = 7)	33% (*n* = 10)
	Private rent	7% (*n* = 2)	7% (*n* = 2)
Household composition	Lives Alone	30% (*n* = 9)	30% (*n* = 9)
	One other occupant	13% (*n* = 4)	17% (*n* = 5)
	Two other occupants	7% (*n* = 2)	3% (*n* = 1)
Funding source	DFG	7% (*n* = 2)	7% (*n* = 2)
	Local Authority (No DFG ** assessment)	10% (*n* = 3)	23% (*n* = 7)
	Mixed	10% (*n* = 3)	0% (*n* = 0)
	Self	23% (*n* = 7)	13% (*n* = 4)
Adaptation Received	Minor (under £1000)	30% (*n* = 9)	13% (*n* = 4)
	Major (£1000+)	20% (*n* = 6)	37% (*n* = 11)

* A terraced house is usually part of a row of houses. ** DFG (Disabled Facilities Grant) may be accessed via local authorities (councils) to resource housing adaptations so that the home may be adapted to individual needs such as installing a stair lift to manage the stairs or ramps to enter and exit the home.
